# Unlocking the Functional Potential of *Lonicera caerulea*: Chemical Profile, Antioxidant, and *α*-Amylase and *α*-Glucosidase Inhibitory Activities of Extracts from Ripe, Unripe, and Lactofermented Fruits

**DOI:** 10.3390/biom16050673

**Published:** 2026-05-01

**Authors:** Karolina Kaptsiuh, Agata Czyżowska, Anna Otlewska, Tomasz Sozański, Alicja Zofia Kucharska

**Affiliations:** 1 Department of Fruit, Vegetable and Plant Nutraceutical Technology, Wrocław University of Environmental and Life Sciences, Chełmońskiego 37, 51-630 Wrocław, Poland; 2 Institute of Fermentation Technology and Microbiology, Lodz University of Technology, Wólczańska 171/173, 90-530 Łódź, Poland; agata.czyzowska@p.lodz.pl (A.C.); anna.otlewska@p.lodz.pl (A.O.); 3 Department of Preclinical Sciences, Pharmacology and Medical Diagnostics, Faculty of Medicine, Wroclaw University of Science and Technology, Wybrzeże Wyspiańskiego 27, 50-370 Wrocław, Poland; tomasz.sozanski@pwr.edu.pl

**Keywords:** *Lonicera caerulea*, honeysuckle berry, fermentation, fruit extracts, chemical composition, loganic acid, cyanidin 3-*O*-glucoside, antioxidant activity, antidiabetic activity

## Abstract

Honeysuckle berries (*Lonicera caerulea*) represent a valuable source of bioactive compounds, primarily flavonoids, and iridoids. This study compared the chemical composition and in vitro antioxidant and antidiabetic properties of resin-purified extracts from ripe, unripe, and unripe lactofermented honeysuckle berries. Polyphenols and iridoids were identified using UPLC-ESI-qTOF-MS/MS and quantified using HPLC-PDA. A total of 6 anthocyanins, 7 phenolic acids, 9 flavan-3-ols, 8 iridoids, 8 flavonols, 3 flavones, and 1 flavanonol were identified in the extracts. The extract from ripe fruits was characterized by a high cyanidin glycoside content (273.59 mg/g) and high iridoid content (138.30 mg/g). The amount of individual iridoids varied among the extracts, with the highest level of loganic acid detected in the unripe fruit extract (39.42 mg/g) and the highest level of sweroside in the ripe fruit extract (55.59 mg/g). Phenolic acid content was approximately twofold higher in extracts from unripe and fermented fruits compared with ripe fruit extracts, suggesting a decrease during ripening, while fermentation did not significantly affect phenolic acid content. Among flavonols, quercetin and isorhamnetin derivatives were identified, with quercetin 3-*O*-rutinoside being the predominant compound in all extracts. The ripe fruit extract exhibited the strongest radical scavenging activity (in ABTS and DPPH assays), ferric ion-reducing power (FRAP), and *α*-amylase inhibition, while all extracts exhibited comparable *α*-glucosidase inhibition. These findings indicate that *L. caerulea* extracts, especially from ripe fruits, are a rich source of biologically active compounds with potential relevance for managing oxidative stress and hyperglycemia.

## 1. Introduction

Oxidative stress and redox imbalance are recognized as key contributors to the pathogenesis of noncommunicable diseases (NCDs), as they promote damage to cellular macromolecules, particularly proteins, ultimately disrupting normal cellular function and homeostasis [1]. NCDs comprise a group of chronic disorders, including cancer, diabetes, cardiovascular diseases, and neurodegenerative conditions. According to estimates from the World Health Organization (WHO), NCDs account for approximately 41 million deaths each year, including around 17 million premature deaths occurring before the age of 70 years [2]. The WHO aims to reduce risk factors for NCDs, which include insufficient physical activity, tobacco use, harmful use of alcohol, and consumption of a Western-type diet [2,3].

The consumption of fruits and vegetables as well as adherence to plant-based dietary patterns are associated with reductions in oxidative stress biomarkers [3,4]. Increased fruit and vegetable intake decreases malondialdehyde (MDA) and oxidized low-density lipoprotein cholesterol (oxLDL-C) levels, while improving plasma total antioxidant capacity (TAC) [3]. The beneficial effects of plant consumption are attributed to the presence of antioxidant vitamins (C and E) and biologically active compounds, including polyphenols and iridoids [1,3,5,6]. Honeysuckle berries (*Lonicera caerulea* var. kamtschatica) are an example of fruits rich in health-promoting bioactive compounds. *L. caerulea* fruits are a valuable source of anthocyanins, quercetin derivatives, iridoids, as well as flavan-3-ols [7].

To date, numerous studies have demonstrated the antioxidant activity of ripe honeysuckle berries in both in vitro [7,8] and in vivo models [9]. The potential use of honeysuckle berries has also been explored in the prevention and alleviation of inflammation [9], cardiovascular diseases [9], and diabetes [10]. The antidiabetic activity may result, among other mechanisms, from the ability of bioactive compounds present in honeysuckle berry fruits to inhibit carbohydrate-digesting enzymes, such as *α*-amylase and *α*-glucosidase [11]. This inhibitory effect is likely associated with their high anthocyanin content [12]. Zhang et al. [12] revealed that anthocyanins isolated from fully ripe honeysuckle berries effectively inhibited *α*-glucosidase activity.

Although the chemical composition, antioxidant activity, and inhibitory effects on *α*-amylase and *α*-glucosidase of ripe honeysuckle berry fruits have been well documented, the bioactive compound profile and biological properties of unripe fruits lacking anthocyanins remain unclear. The stage of fruit maturity significantly influences the profile of biologically active compounds and, consequently, the biological activity of fruits. Previous studies on the chemical composition of other fruits, such as blueberries, have shown that despite the absence of anthocyanins, these fruits are rich in polyphenolic compounds, particularly flavonols, phenolic acids, and flavan-3-ols, including catechin and epicatechin [13].

In addition to fruit maturity, fermentation represents a separate factor that may significantly influence the chemical composition and health-promoting properties of fruits. Lactic acid fermentation of unripe fruits is used in food technology, for example, during the production of green table olives (also known as Spanish-style olives) [14]. Another example of lactofermented unripe fruits is fermented cornelian cherry, a traditional product of southeastern Poland that has historically been consumed as a table olive substitute [15]. It is expected that fermented fruits may exhibit a different chemical composition and biological activity, as bacterial enzymatic activity during fermentation can degrade high-molecular-weight compounds, including tannins [16], while also increasing antioxidant activity [17,18]. It has been previously reported that lactic acid bacteria (LAB) may also reduce phenolic acids, for example, converting caffeic acid into dihydrocaffeic acid, or decarboxylate hydroxycinnamic acids into the corresponding vinyl derivatives and subsequently into ethyl compounds [19]. LAB also reduce flavonols (both glycosides and aglycones) [17] and hydrolyze iridoid glycosides [20]. As the ability of extracts to inhibit carbohydrate-digesting enzymes is typically correlated with their bioactive compound profile [8,21], differences in this activity between unripe and fermented fruits may be expected.

To the best of our knowledge, no studies conducted by other research groups have investigated the lactic acid fermentation of unripe honeysuckle berries, and consequently, the chemical composition and biological properties of the fermented fruits remain undetermined.

This study aimed to compare the chemical composition, as well as in vitro biological properties of extracts obtained from ripe, unripe, and unripe lactofermented honeysuckle berries. Particular attention was given to the impact of fruit maturity and fermentation on the profile of bioactive compounds in the extracts, their antioxidant activity, and ability to inhibit carbohydrate-digesting enzymes. This study reports, for the first time, the development of an innovative food product: lactofermented honeysuckle berries. The fermentation process was conducted using the starter cultures *Levilactobacillus brevis* SBHE2 and *Lactiplantibacillus plantarum* ZP71, which was applied for the production of a fermented food for the first time. The use of two strains allows us to determine which one is more suitable for the preparation of fermented honeysuckle berries.

## 2. Materials and Methods

### 2.1. Chemicals

Potassium persulfate, 2,2′-azinobis(3-ethylbenzothiazoline-6-sulfonic acid) (ABTS), 1,1-diphenyl-2-picrylhydrazyl (DPPH), 6-hydroxy-2,5,7,8-tetramethylchroman-2-carboxylic acid (Trolox), 2,4,6-tri(2-pyridyl)-s-triazine (TPTZ), ferrous chloride, and *p*-nitrophenyl-*α*-D-glucopiranoside were supplied by Sigma Chemicals Co. (Steinheim, Germany). Formic acid (HPLC grade), *α*-amylase from porcine pancreas, and intestinal acetone powders from rat were purchased from Sigma-Aldrich (St. Louis, MO, USA). Calcium chloride was purchased from Eurochem BGD (Tarnów, Poland). Methanol and acetonitrile (both HPLC grade), as well as hydrochloric acid, were obtained from POCh (Gliwice, Poland). Acetic acid, sodium acetate, and sodium carbonate were supplied by Chempur (Piekary Śląskie, Poland). All reference standards were of HPLC purity grade. Loganic acid, sweroside, and cyanidin 3-*O*-glucoside were obtained from PhytoLab (Phyproof Reference Standards, Vestenbergsgreuth, Germany); loganin was purchased from Supelco (Bellefonte, PA, USA); 3-*O*-, and 5-*O*-caffeoylquinic acids were supplied by TRANS MIT (Gießen, Germany); and quercetin 3-*O*-glucoside and quercetin 3-*O*-rutinoside were obtained from Extrasynthese (Genay, France).

### 2.2. Plant Material

Honeysuckle berries were harvested in Huwniki, Poland (49°39′18″ N, 22°42′18″ E) in 2023. The fruits were collected at two stages of maturity: unripe, when the berries were completely green with no purple coloration, and fully ripe, when the berries were soft, purple, and covered with a white wax coating. Unripe fruits were collected in May, while ripe fruits were collected in June.

### 2.3. Fermentation

Fresh unripe honeysuckle berries were fermented in single batches in 2% brine. The products differed in fermentation location (Institute of Fermentation Technology and Microbiology, Łódź, Poland or Arboretum and Institute of Physiography in Bolestraszyce near Przemyśl, Poland), type of fermentation (spontaneous or with the addition of a starter culture), fermentation temperature, and the use of dried spices (none, savory, or thyme; 1.3 g/L). Six products were prepared: 1—product prepared from fresh honeysuckle berries with the addition of thyme, fermented under home-made conditions and spontaneously (fresh fruits + T (HM, SF)); 2—product prepared from fresh honeysuckle berries with the addition of savory, fermented under home-made conditions and spontaneously (fresh fruits + S (HM, SF)); 3—product prepared from frozen honeysuckle berries with the addition of thyme, fermented under home-made conditions and spontaneously (frozen fruits + T (HM, SF)); 4—product prepared from fresh honeysuckle berries with the addition of thyme, fermented at 17 °C and spontaneously (fresh fruits + T (17 °C, SF)); 5—product prepared from fresh honeysuckle berries with the addition of thyme, fermented at 17 °C under controlled conditions (CF) with starter culture *Lactiplantibacillus plantarum* ZP71 (fresh fruits + T (17 °C, CF—*Lactiplantibacillus plantarum* ZP71)); 6—product prepared from fresh honeysuckle berries with the addition of thyme, fermented at 17 °C under controlled conditions (CF) with starter culture *Levilactobacillus brevis* SBHE2 (fresh fruits + T (17 °C, CF—*Levilactobacillus brevis* SBHE2)). After 90 days of fermentation, microbial analysis and analysis of basic fermentation by-products were performed. Due to their sugar content, ripe fruits were not subjected to lactic acid fermentation, as this could promote alcoholic followed by acetic fermentation, resulting in a fundamentally different product (e.g., wine or vinegar).

The starter strains were previously isolated from lactofermented green tomato and beetroot at the Institute of Fermentation Technology and Microbiology, Łódź, Poland. The 16S rRNA genes of *Lactiplantibacillus plantarum* ZP71 and *Levilactobacillus brevis* SBHE2 were isolated and analyzed according to the method described by Bernacka et al. [22]. The 16S rRNA gene sequences corresponding to *L. plantarum* ZP71 and *L. brevis* SBHE2 were submitted to GenBank and assigned the accession numbers PX444863 and PX444864, respectively.

### 2.4. Microbial Analysis

Microbiological analysis was performed according to ISO 6887-1:2017 (ISO, Geneva, Switzerland) [23]. All culture media were purchased from Merck KGaA (Darmstadt, Germany). Enterobacteriaceae were enumerated on Violet Red Bile Dextrose agar after incubation at 37 °C for 48 h. Total mesophilic count was determined on Plate Count Agar after incubation at 30 °C for 48 h. LAB were cultured on Man, Rogosa and Sharpe agar supplemented with nystatin (Ambeed, Buffalo Grove, IL, USA; 3 mL of 0.21% stock solution per 100 mL of medium) and incubated at 30 °C for 48 h. Yeasts and molds were enumerated on Dichloran Rose Bengal Chloramphenicol agar after incubation at 30 °C for 48 h.

Based on results of microbial analysis, one product was selected for extract preparation. The absence of bacteria from the family Enterobacteriaceae and molds, along with the presence of LAB, was considered a necessary criterion in the selection of the product for extract preparation. Accordingly, the product selected for further analysis was prepared with the addition of thyme, inoculated with the *Levilactobacillus brevis* SBHE2 starter strain, and fermented under controlled temperature conditions (17 °C). The term ‘fermented fruit extract’ is used in the rest of the manuscript as a simplified designation for extract from this product.

### 2.5. Fruit Extract Preparation

Before extract preparation, ripe, unripe, and fermented fruits were stored at −20 °C. Extracts were prepared according to the protocol described previously by Bernacka et al. [22]. Frozen fruits were heated in a Thermomix (Vorwerk, Wuppertal, Germany) for 3 min at 95 °C with the addition of 1% citric acid solution. After cooling to 50 °C, a depectinizing enzyme (Pectinex Ultra Color; Novozymes, Bagsværd, Denmark) was added at a concentration of 1 g per 1 kg of fruit. Depectinization was carried out at 50 °C for 2 h under controlled temperature conditions. Subsequently, the fruits were pressed using a hydraulic press (SRSE, Warsaw, Poland).

The obtained juices were purified using a polymeric adsorbent, Amberlite XAD-16 resin (Rohm and Haas, Chauny Cedex, France). Sugars and organic acids were removed by washing the resin with distilled water, followed by elution of phenolic compounds with 80% (*v*/*v*) aqueous methanol. The methanolic fractions were evaporated under reduced pressure at 40 °C (Büchi, Flawil, Switzerland) and subsequently lyophilized (Alpha 1–4 LSC, Martin Christ Gefriertrocknungsanlagen GmbH, Osterode am Harz, Germany). Extraction yield varied by raw material. From 100 g of frozen fruit, 1.4, 4.1, and 1.2 g of lyophilized extract were obtained, respectively. The resulting powdered extracts were stored at −20 °C until further analysis. The results reported in Section 3.2, Section 3.3, Section 3.4, Section 3.5 and Section 3.6 refer to processed and purified extracts rather than the native fruit matrix.

### 2.6. Identification of Phenolic Compounds and Iridoids by UPLC-ESI-qTOF-MS/MS

Before analysis, the extracts were dissolved in 80% MeOH containing 1% HCl and filtered through membrane filters with a pore size of 0.22 µm (Chemland, Stargard, Poland). Analysis was performed using an Acquity ultra-performance liquid chromatography system coupled with a quadrupole time-of-flight mass spectrometer (UPLC/Synapt Q-TOF MS; Waters Corp., Milford, MA, USA). Separation was achieved on a C18 column (100 × 2.1 mm i.d., 1.7 µm; Acquity UPLC BEH, Waters Corp., Milford, MA, USA) according to the protocol described previously by Przybylska et al. [24]. Anthocyanins were identified in the positive ion mode, whereas the remaining polyphenols and iridoids were identified in the negative ion mode. Data acquisition was performed using MassLynx software (v. 4.1; Waters Corp., Milford, MA, USA).

### 2.7. Quantification of Phenolic Compounds and Iridoids by HPLC-PDA

Extracts were dissolved in 80% MeOH containing 1% HCl and filtered through membrane filters with a pore size of 0.45 µm (Chemland, Stargard, Poland). HPLC analysis was carried out using a Dionex system (Germering, Germany) equipped with a diode array detector (Ultimate 3000) according to the protocol previously described by Przybylska et al. [24]. Separation was performed on a C18 column (250 × 4.6 mm, 5 µm; Hypersil GOLD C18; Thermo Fisher Scientific Inc., Dartford, UK). Data acquisition was performed using Chromeleon 7.2 software (Thermo Scientific Dionex, Sunnyvale, CA, USA). The results are expressed as the mean of three replicates ± standard deviation and expressed as mg per g of freeze–dried extract.

### 2.8. Determination of Total Phenolic Content and Antioxidant Activity In Vitro

Total phenolic content (TPC) was determined using the Folin–Ciocalteu method [25], and antioxidant activity was measured using the ABTS [26], DPPH [27], and FRAP [28] assays. All methods were performed with slight modifications, as described by Przybylska et al. [24]. Absorbance was measured using a microplate reader (Synergy H1, BioTek, Winooski, VT, USA). Before each analysis, the extracts were dissolved in 80% MeOH containing 1% HCl and analyzed in two replicates. Each solution was applied to 96-well plates in four repetitions. Data are expressed as the mean ± standard deviation and reported as g of gallic acid equivalents per 100 g of freeze–dried extract (TPC) and mmol Trolox (Tx) equivalents per 100 g of freeze–dried extract (antioxidant assays).

### 2.9. Determination of Antidiabetic Activity In Vitro

*α*-Amylase and *α*-glucosidase inhibitory activities were determined according to the protocol previously described by Podsędek [29]. Absorbance was measured using a microplate reader (Synergy H1, BioTek, Winooski, VT, USA). Before the experiment, the extract solutions (in 80% MeOH containing 1% HCl) were prepared in two replicates. Each measurement, for both the extract and blank, was performed in four replicates. IC_50_ values were calculated from linear regression curves based on three extract concentrations (4, 2 and 1 mg/mL for α-amylase and 2, 1 and 0.5 mg/mL for α-glucosidase). Results are expressed as IC_50_ values ± standard deviation.

### 2.10. Statistical Analysis

Statistical analysis was performed using Statistica 14.0.0.15 (TIBCO Software Inc., Palo Alto, CA, USA), applying one-way ANOVA followed by Duncan’s post hoc test to identify significant differences between groups and Pearson’s correlation analysis. Principal component analysis (PCA) was performed using PAST 4.12 (Natural History Museum, University of Oslo, Oslo, Norway).

## 3. Results and Discussion

### 3.1. Microbial Analysis

The microbiota in unripe fermented *L. caerulea* fruits are summarized in Table 1. The total mesophilic bacteria counts in the tested samples ranged from 10^5^ to 10^7^ CFU/mL. LAB counts exceeded 1 log CFU/mL only in sample 6. As the microbiota was assessed at a single time point, high LAB count may have been present in products 1–5 during earlier stages of fermentation. Yeast counts in samples 1–5 were approximately 10^5^ CFU/mL. Sample 6, fermented with *Levilactobacillus brevis* SBHE2, was characterized by the lowest yeast count (1.48 log CFU/mL). The count of Enterobacteriaceae in all samples does not exceed 1 log CFU/mL. The absence of bacteria from the Enterobacteriaceae family, the lack of LAB in most samples, and the high abundance of yeasts may be related to the fact that, in the case of fermented unripe berries, the fermentation process may have already reached a late stage. Previous studies on olive fermentation have shown that Enterobacteriaceae are typically present during the initial stages of fermentation; subsequently, the environment gradually acidifies, as LAB develop, leading to a phase dominated by LAB [30]. At later stages of fermentation, white colony-forming yeasts may proliferate, which have been associated with a deterioration in product quality. In our previous study [22], unripe cornelian cherry fruits were used as a raw material to fermentation. Similar to the present results for honeysuckle berries, LAB were detected only in samples inoculated with a starter culture (*Levilactobacillus brevis* ZP HE1), whereas in non-inoculated samples the LAB count remained below 1 log CFU/mL. The microbial community analysis in fermented cornelian cherry fruits was likely performed at a late stage of fermentation, while the presence of lactic acid in all samples suggested that LAB had been active during the earlier stages of the process [22]. A similar phenomenon may also have occurred during honeysuckle berry fermentation without starter cultures.

### 3.2. Identification of Polyphenols and Iridoids in Extracts

**Figure 1:**
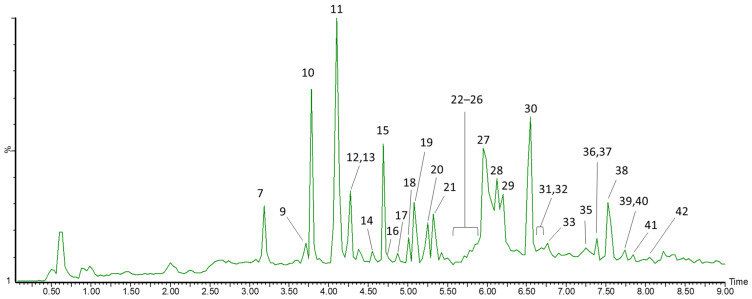
Total ion chromatogram (TIC) of the extract of unripe honeysuckle berry fruits obtained by UPLC-ESI-qTOF-MS/MS. Peak numbers in the chromatogram correspond to the compound numbers listed in Table 2.

The total ion chromatogram (TIC) of the unripe honeysuckle berry extract is shown below (Figure 1). Table 2 presents the UPLC-ESI-qTOF-MS/MS analysis of resin-purified extracts from ripe, unripe, and unripe lactofermented *L. caerulea* fruits. The compounds were identified based on their retention times, elution order, literature data, [M − H]^−^ and [M + H]^+^ ions, as well as fragment ions obtained in MS/MS analysis. Anthocyanins were identified in positive ion mode, whereas other phenolic compounds and iridoids were identified in negative ion mode. In the present study, we identified 6 anthocyanins, 7 phenolic acids, 9 flavan-3-ols, 8 iridoids, 8 flavonols, 3 flavones, and 1 flavanonol.

**Figure 2:**
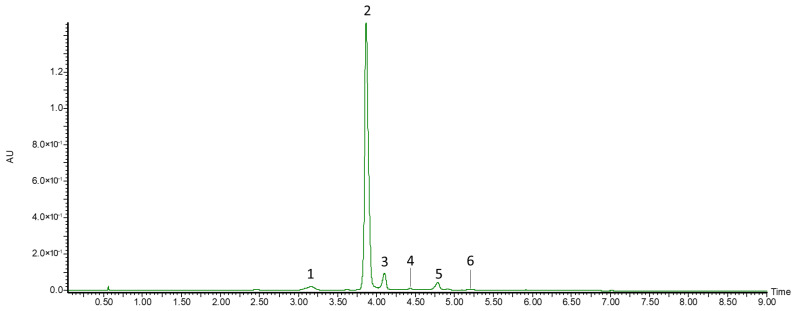
UPLC-PDA chromatogram of anthocyanins identified in the extract of ripe honeysuckle berry fruits recorded at λ = 520 nm. Peak numbers in the chromatogram correspond to the compound numbers listed in Table 2.

Among the anthocyanins, three monoglycosides and three diglycosides were identified (Figure 2). Compound **1**, which showed an [M + H]^+^ ion and fragment ions at *m*/*z* 449 and 287, was identified as cyanidin-3,5-*O*-diglucoside. Compounds **2** and **3** showed pseudomolecular ions at *m*/*z* 449 and 595, respectively, and both produced a fragment ion at *m*/*z* 287, corresponding to cyanidin; therefore, they were identified as cyanidin-3-*O*-glucoside and cyanidin-3-*O*-rutinoside, respectively. Compounds **4**–**6** showed pseudomolecular ions at *m*/*z* 433, 463, and 597, respectively, and were identified as pelargonidin-3-*O*-glucoside, peonidin-3-*O*-glucoside, and delphinidin-3-*O*-sambubioside. All identified anthocyanins have previously been reported in *L. caerulea* fruits [7,31,32,33,34]. Guo et al. [33] identified a total of 14 anthocyanin glycosides in honeysuckle berry fruits, including additional cyanidin, peonidin, and delphinidin derivatives, such as cyanidin-3-(6″-acetyl)-glucoside, cyanidin-3-sambubioside, peonidin-3-glucoside pyruvic derivative, and delphinidin-3-rutinoside.

**Figure 3:**
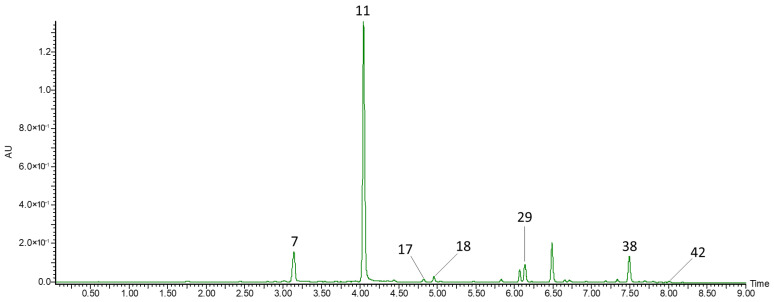
UPLC-PDA chromatogram of phenolic acids identified in the extract of unripe honeysuckle berry fruits recorded at λ = 320 nm. Peak numbers in the chromatogram correspond to the compound numbers listed in Table 2.

Compounds **7**, **11**, **17**, **18**, **29**, **38**, and **42** were identified as phenolic acids (Figure 3). Compounds **7**, **11**, and **18** gave pseudomolecular ions at *m*/*z* 353 and fragment ions at *m*/*z* 191 ([M−caffeoyl]^−^) and, based on their elution order, were identified as 3-*O*-caffeoylquinic acid, 5-*O*-caffeoylquinic acid, and a caffeoylquinic acid isomer, respectively. Compounds **38** and **42** gave a pseudomolecular ion at *m*/*z* 515 and a fragment ion at *m*/*z* 353, which is characteristic of dicaffeoylquinic acids. Caffeoylquinic acid derivatives are characteristic of ripe honeysuckle berry fruits [7,21,31,32,34,35,39,40], as well as of other berries such as cranberries [41] and lingonberries (*Vaccinium vitis-idaea* L.) [42].

Compounds **17** and **29** gave pseudomolecular ions at *m*/*z* 367 and were identified as caffeoylquinic acid methyl ester isomers. Caffeoylquinic acid methyl ester (1) gave a fragment ion at *m*/*z* 161 ([caffeic acid − H − H_2_O]^−^), whereas *O*-caffeoylquinic acid methyl ester (2) gave two fragment ions at *m*/*z* 179 ([caffeic acid − H]^−^) and 135 ([caffeic acid − H − CO_2_]^−^). Caffeoylquinic acid methyl ester has previously been detected in *Lonicera japonica* [37,38] and *Lonicera syringantha* flos [38].

Compound **8** showed a pseudomolecular ion at *m*/*z* 627 and two characteristic fragment ions at *m*/*z* 303 and 285 in negative ion mode, corresponding to taxifolin aglycone and dehydrated taxifolin aglycone ([aglycone − 18]^−^), respectively. This compound was the only flavanone (dihydroflavonol) identified in the present study and was detected exclusively in ripe fruit extract. Taxifolin 7-*O*-dihexoside has previously been reported in ripe honeysuckle berry fruits although its identification was performed in positive ion mode [7]. Taxifolin glycosides have also been reported in other fruits, including, rose hip berry (*Rosa canina*) [43,44], and alcoholic fermented strawberry products [45].

Compound **19** showed a pseudomolecular ion at *m*/*z* 289 and a characteristic fragment ion at *m*/*z* 245 after loss of CO_2_ ([epicatechin − 44]^−^), and was tentatively identified as epicatechin. Epicatechin was abundant in all examined extracts and has previously been reported in ripe honeysuckle berries by [7,35,39]. Besides epicatechin, two procyanidin dimers (**9**, **37**), four trimers (**16**, **20**, **22**, **31**), and two tetramers (**13**, **25**) with characteristic pseudomolecular ions at *m*/*z* 577, 865, and 1153, respectively, were observed. Procyanidin oligomers showed characteristic fragmentation patterns, as previously reported by Wojdyło et al. [35]. All nine flavan-3-ols were abundant only in extracts from unripe honeysuckle berries. In ripe fruit and fermented fruit extracts, epicatechin and procyanidin dimers remained abundant, whereas fewer trimer and tetramer isomers were detected.

Among flavonols, five quercetin derivatives (**24**, **28**, **30**, **33**, **40**) and three isorhamnetin derivatives (**34**, **36**, **39**) were identified in *L. caerulea* extracts. The quercetin and isorhamnetin derivatives gave fragment ions at *m*/*z* 301, and 315, respectively, corresponding to their aglycones. All flavonols, with the exception of isorhamnetin hexoside-pentoside (**34**) and isorhamnetin hexoside (**39**), have been previously reported in honeysuckle berry fruits [7,21,31,35,39].

**Figure 4:**
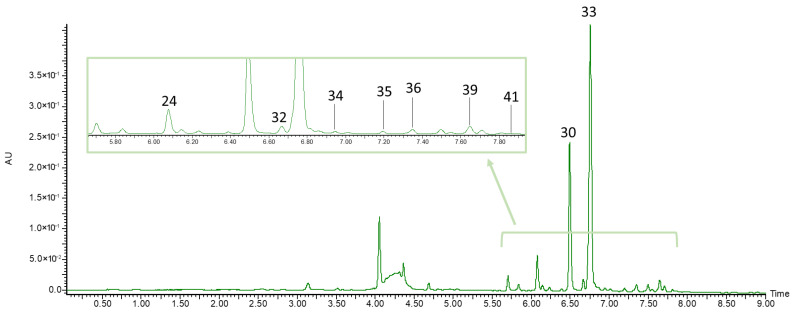
UPLC-PDA chromatogram of flavonols and flavones identified in the extract of ripe honeysuckle berry fruits recorded at λ = 360 nm. Peak numbers in the chromatogram correspond to the compound numbers listed in Table 2.

In all fruit extracts, three flavones were identified (**32**, **35** and **41**). Compound **32** gave a pseudomolecular ion at *m*/*z* 593 and two fragment ions at *m*/*z* 447 and 285, corresponding to [M − rhamnose]^−^ and the luteolin aglycone, respectively. Compound **41** gave a pseudomolecular ion at *m*/*z* 607 and a fragment ion at *m*/*z* 299, corresponding to the diosmetin aglycone. The flavonols and flavones identified in the extract of ripe honeysuckle berry fruits are presented in Figure 4.

Compounds **10**, **12**, **15**, **21**, **23**, and **26** belong to iridoids, a non-phenolic group of biologically active compounds occurring in honeysuckle berries. In the current study, we identified loganic acid (**10**) and its derivatives (**12**, **15**), pentosyl loganin (**21**), sweroside (**23**), and its derivative (**26**). All of the above-mentioned iridoids have been previously reported in *L. caerulea* fruits. The fragmentation patterns and plausible structures of these compounds were thoroughly characterized by Kucharska and Fecka [36]. In the current study, we also identified an oxidized form of loganic acid—dehydrologanic acid (**14**), which gave a pseudomolecular ion at *m*/*z* 373 ([loganic acid − 2H]^−^). The fragment ions of dehydrologanic acid were also characterized by a loss of 2 Da compared with non-oxidized loganic acid, yielding fragment ions at *m*/*z* 211, 167, and 149.

### 3.3. Quantification of Polyphenols and Iridoids in Extracts

In extracts from ripe, unripe, and unripe lactofermented honeysuckle berries, the content of individual polyphenols as well as iridoids was determined by the HPLC-PDA method (Table 3). In the present study, the concentrations of polyphenols and iridoids in the extracts were higher than those in the fresh or dry fruit material [7,31,35,39] because the extracts were purified using a polymeric adsorbent resin to remove sugars and organic acids and subsequently freeze-dried. As a result, the extracts constituted a concentrated source of bioactive compounds.

In the ripe fruit extract, anthocyanins constituted the highest proportion of the quantified compounds (59%), followed by iridoids (28%) and phenolic acids (8%). In the unripe fruit extract, two groups of compounds showed comparable contributions, namely iridoids (46%) and phenolic acids (43%). In contrast, phenolic acids predominated in the extract from fermented fruits (47%). Flavonols represented the lowest proportion among the quantified compounds in all analyzed extracts.

#### 3.3.1. Anthocyanins

Anthocyanin content in the ripe fruit extract reached 289.72 mg/g of extract, exceeding the levels reported for Amberlite XAD-16 resin-purified extracts from other anthocyanin-rich crops, including red cabbage (175.00 mg/g) and purple carrot (154.44 mg/g) [46]. In contrast, the extract from lingonberry purified on Amberlite XAD-7 was characterized by low anthocyanin content (24.40 mg/g) [42].

In the ripe fruit extract, the predominant anthocyanin was cyanidin 3-*O*-glucoside, which is characteristic of the anthocyanin profile of honeysuckle berries reported by other authors [7,21,31,35,39]. Pelargonidin, peonidin, and other cyanidin derivatives were detected in lower amounts.

#### 3.3.2. Phenolic Acids

In extracts from unripe and unripe lactofermented fruits, the total amount of phenolic acids was comparable and more than twofold higher than in the ripe fruit extract. The total phenolic acid content determined in ripe honeysuckle berry extract (37.37 mg/g) was lower than that reported for resin-purified red cabbage extract (41.03 mg/g) [46] and markedly higher than values reported for cornelian cherry extracts (3.89 mg/g) [47]. A decrease in phenolic acid content during fruit ripening has also been observed in other fruits, including lingonberry [48], apples [49], and cornelian cherry (*Cornus mas* L.) [50]. In *V. vitis-idaea*, the content of cinnamic acid derivatives decreased threefold during ripening. The predominant cinnamic acid derivative in unripe fruits was a ferulic acid-hexoside isomer, and its content decreased 39-fold during ripening. The content of chlorogenic acid in unripe lingonberries was low and decreased only twofold compared with fully ripe fruits [48]. In contrast, Fecka et al. [51] reported that in strawberries, the total content of cinnamic acid derivatives, calculated as the sum of 1-*O*-trans-cinnamoyl-*β*-glucose and 1-*O*-p-coumaroyl-*β*-glucose, increased 12.4-fold during fruit ripening. An increase in the content of 1-*O*-trans-cinnamoyl-*β*-glucose and 1-*O*-*p*-coumaroyl-*β*-glucose during strawberry ripening was also previously reported by Latza et al. [52]. The increase in their content may be related to their role as precursors of hydroxycinnamoyl esters and volatile cinnamates [52].

In the current study, the content of 5-*O*-caffeoylquinic acid was higher in the unripe fermented fruit extract than in the unripe fruit extract (65.31 vs. 56.10 mg/g). It has been reported that LAB may metabolize 5-*O*-caffeoylquinic acid during fermentation, releasing free caffeic and quinic acids. Caffeic acid may subsequently be converted to dihydrocaffeic acid or vinyl catechol, whereas quinic acid may be transformed into shikimic acid and further reduced to dihydroshikimic acid [19]. Quinic acid may be advantageous during fermentation because it can replace pyruvate and fructose as a hydrogen acceptor and serve as an energy source for heterofermentative bacteria in substrates poor in fructose, such as vegetables or unripe fruits [19].

#### 3.3.3. Flavonols

Total flavonol content in honeysuckle berry extracts ranged between 20.83 and 27.53 mg/g of extract. In contrast, resin-purified extracts from cornelian cherry fruits contained lower amount of flavonols (5.89 mg/g of extract) [47]. In all examined extracts, quercetin-3-*O*-glucoside was the most abundant flavonol, followed by quercetin-3-*O*-rutinoside. Isorhamnetin glycosides was present in all examined extracts in lower amounts. The flavonol profile of four cultivars of ripe honeysuckle berries was analyzed by Senica et al. [39]. In the study conducted by Senica et al. [39] quercetin 3-*O*-rutinoside was the predominant compound, followed by quercetin 3-*O*-galactoside and quercetin 3-*O*-acetyl-hexoside [39].

In the current study, the contents of quercetin-*O*-vicianoside, quercetin-*O*-rutinoside, and isorhamnetin 3-*O*-rutinoside were significantly lower in ripe fruit extract than in unripe fruit extract, whereas the total flavonol content was 1.2-fold higher in ripe fruit extract. These results contrast with those reported by Li et al. [53], who observed a sixfold decrease in total flavonol content during ripening of chinese raspberry fruits (*Rubus chingii* Hu), from the fully green stage to the red stage. This discrepancy may be related to differences in fruit anatomical structure. In *R. chingii*, kaempferol and quercetin derivatives are characteristic compounds of epidermal hairs and fruit placentae, anatomical structures that are not abundant in honeysuckle berry [53].

The total flavonol content was significantly higher in extracts from unripe fermented berries than in those from unripe berries. Quercetin 3-*O*-glucoside levels were also significantly higher in extract from fermented fruits compared with the other extracts. This increase in flavonol glycosides, including quercetin glucoside, may be partially attributed to thyme addition during fermentation, as thyme is a source of quercetin glycosides [54].

#### 3.3.4. Iridoids

The loganic acid content was the highest in the unripe fruit extract. Loganic acid has previously been reported as the predominant iridoid in ripe fruits of 30 honeysuckle berry cultivars, and its content in the cultivar ‘Wojtek’ was 100.13 mg/100 g fresh weight (fw) [7] which is about 20-fold lower than in the resin-purified ripe fruit extract. In the current study, loganic acid content decreased during fruit ripening and the fermentation of unripe fruits. The ripe fruit extract was characterized by a high content of sweroside (55.92 mg/g extract), as well as loganic acid derivatives, including loganic acid 7-*O*-pentoside and 7-epi-loganic acid 7-*O*-pentoside. Kucharska et al. [7] reported that in the honeysuckle berry cultivar ‘Wojtek’, the sum of sweroside and loganin was significantly higher than in the remaining 29 cultivars, suggesting that a high sweroside content may be characteristic of this cultivar.

The total iridoid content in ripe fruit extracts was about 1.5 times higher than that in unripe fruit extracts. Despite the increase in total iridoid content after fruit ripening, the content of loganic acid significantly decreased. In contrast, Przybylska et al. [50] reported that in four cultivars of *C. mas*, both the total iridoid content and content of the two predominant iridoids, loganic acid and cornuside decreased during ripening. Additionally, a decrease in loganin content during fruit maturation has also been reported in *Cornus officinalis* fruits [55]. Changes in the content of selected iridoids in honeysuckle berry extracts may be associated with the biosynthetic pathway of iridoids. Loganic acid acts as a precursor of loganin, which is subsequently converted to secologanin, the direct substrate for sweroside biosynthesis [56]. Thus, loganic acid present in unripe honeysuckle berries may be partially transformed into sweroside, explaining the decrease in loganic acid content accompanied by an increase in sweroside content during ripening.

In the current study, the loganic acid content in the fermented fruit extract was lower than that in the unripe fruit extract. Because iridoids are uncommon in food products [57], information on their profile and content in fermented foods remains limited. During lactic acid fermentation of olives, the content of secoiridoids, including oleuropein and ligstroside, decreases. The degradation of iridoids during fermentation occurs due to the *β*-glucosidase activity of LAB [58]. A similar phenomenon has been reported for the degradation of the iridoid glycoside geniposide during fermentation of gardenia fructus (*Gardenia jasminoides* J. Ellis) extract with the LAB starter culture *Lactobacillus plantarum* SN13T [59].

### 3.4. TPC and Antioxidant Activity

The TPC and antioxidant activity of *L. caerulea* extracts and standards are shown in Table 4. The TPC values obtained for the extracts in the current study are relatively high, which is likely a result of the use of resin-purified extracts for the analyses and the expression of the results per lyophilized extract. The TPC was the highest in the anthocyanin-rich extract obtained from ripe fruits (39.01 g GAE/100 g of extract). The TPC of the lingonberry fruit extract, purified using an Amberlite XAD-7 resin, was higher than that of honeysuckle berry extract and exceeded 50 g/100 g [42], while the TPC of the cornelian cherry fruit extract purified on Amberlite XDD-16 resin was lower (25.9 g/100 g) [22]. The TPC in extracts obtained from unripe cornelian cherry fruits was higher than that in unripe honeysuckle berry extracts (51.4 vs. 22.91 g/100 g), which may be attributed to the high content of hydrolysable tannins in unripe cornelian cherry fruits. Similarly to extracts obtained from unripe cornelian cherry fruits, the TPC value in fermented unripe honeysuckle berry extracts was lower than that in raw green fruits, which may result from the hydrolysis of complex compounds by bacterial enzymes during fermentation [22].

In our study, extracts from unripe honeysuckle berries exhibited a 1.7-fold lower TPC than extracts obtained from ripe fruits. The increase in TPC in dark-colored fruits during ripening has been previously reported for blueberries (*Vaccinium* spp.) [60] and blackberries (*Rubus* spp.) [61]. Hwang et al. [60] reported that unripe blueberry fruits exhibited a 1.3-fold lower TPC than ripe fruits. However, in that study, unripe fruits were defined as those with a low anthocyanin content (19.67–40.28 mg/100 g fw), whereas in our study, unripe fruits were harvested at an earlier stage of development, when no anthocyanins were present. The fermentation of unripe honeysuckle berries did not significantly affect the TPC. Although there are no literature data regarding TPC in fermented whole honeysuckle berries, Liang et al. [62] reported a significant decrease (*p* < 0.05) in TPC of honeysuckle berry juice after 24 h of fermentation with the starter strain *Lacticaseibacillus rhamnosus* L08. The difference may be due to variations in the content of reducing sugars in unripe fruit and ripe berry juice. Ripe honeysuckle berry juice before fermentation may contain high levels of reducing sugars, such as fructose and glucose, which, like polyphenols, can reduce the Folin–Ciocalteu reagent [63]. The TPC in juice may decrease after fermentation with *L. rhanmosus* L08 because these sugars are metabolized by bacteria. In our study, we used unripe berries, which contain less glucose and fructose [40], and this may explain why TPC did not change significantly after fermentation.

Cyanidin-3-*O*-glucoside exhibited the highest antioxidant activity in all three assays (ABTS, DPPH, and FRAP), followed by the anthocyanin-rich extract from ripe fruits. Previously, high antioxidant activity of cyanidin-3-*O*-glucoside in the ABTS assay (about 450 mmol Tx/100 g), significantly higher (*p* < 0.05) than that of reference compounds (Tx and vitamin C), was reported [64].

The antioxidant activity of anthocyanin-rich ripe honeysuckle berry extract measured by the ABTS method was higher than that of the resin-purified extract from red cabbage (253 mmol Tx/100 g) and lower than that of the resin-purified extract from purple carrot (383 mmol Tx/100 g) and resin-purified passion fruit (*Passiflora edulis* Sims) epicarp extract (1004.40 mmol Tx/100 g). Similarly, in the DPPH and FRAP assays, ripe honeysuckle berry extract showed lower antioxidant activity than purple carrot extract but higher than red cabbage extract [46].

The antioxidant activity of resin-purified extracts was previously evaluated for ripe, unripe, and lactofermented unripe cornelian cherry fruits. Because anthocyanins were less abundant in the *C. mas* extract obtained from ripe fruits, its antioxidant activity measured by the ABTS assay was lower (222 mmol Trolox/100 g) than that of the anthocyanin-rich honeysuckle berry extract. Extracts from unripe and lactofermented unripe *C. mas* fruits exhibited 3.6- and 3.4-fold higher antioxidant activity, respectively, whereas extracts from unripe and lactofermented honeysuckle berry were less active [22]. The higher radical-scavenging activity of unripe *C. mas* extracts may be attributed to their elevated ellagitannin content. Consistently, ellagitannin fractions isolated from raspberry and cloudberry (*Rubus chamaemorus*) showed more than twofold higher antioxidant activity in the DPPH assay than the corresponding fruit extracts [65].

Although the antioxidant activity of ripe honeysuckle berries has been confirmed in numerous studies [7,21,31,35,66], the antioxidant activity of unripe fruits has not yet been reported. In the present study, we demonstrated that extracts from unripe fruits exhibited lower antioxidant activity than those from ripe fruits. A similar trend during the ripening process has been reported for five blueberry cultivars [60]. Hwang et al. [60] showed that unripe fruits displayed lower antioxidant activity in both ABTS and FRAP assays. In contrast, this tendency has not been confirmed for cornelian cherry fruits [50]. Przybylska et al. [50] monitored the ripening process across six stages of cornelian cherry fruit development and reported that, for four cultivars, antioxidant activity measured by ABTS and FRAP assays decreased. Antioxidant activity is generally correlated with the polyphenol content of fruits [8,21,60]. In honeysuckle berry and blueberry fruits, both TPC and antioxidant activity increase during ripening [60], whereas in cornelian cherry fruits, the phenolic content decreases, which is associated with reduced antioxidant activity [50]. The antioxidant activity of extracts from unripe and unripe fermented honeysuckle berries did not differ significantly, which may suggest that lactic acid fermentation with *Levilactobacillus brevis* SBHE2 did not affect the antioxidant activity of unripe honeysuckle berries.

Although no in vitro antioxidant activity was detected in the present study, the antioxidant potential of loganic acid has been demonstrated in rodent models. In streptozotocin (STZ)-induced diabetic rats, loganic acid supplementation reduced intracellular ROS levels and increased reduced glutathione (GSH) concentration and the activity of catalase (CAT), glutathione peroxidase (GPx), and glutathione reductase (GR) in leukocytes [67]. In a high-fat diet/STZ-induced diabetic mouse model, loganic acid improved redox imbalance by decreasing MDA and increasing GSH levels [68]. Antioxidant activity was also confirmed in a dextran sulfate sodium-induced colitis rat model, where loganic acid supplementation reduced intracellular ROS levels, increased GSH levels in colon tissue, and decreased MDA levels [69]. The in vitro antioxidant assays used in our study are based on simple reaction mechanisms, including hydrogen atom transfer (ABTS) and electron transfer (DPPH, FRAP) [70]. In the studies mentioned above, loganic acid was administered orally; therefore, its antioxidant effects in vivo may result from modulation of genes involved in redox homeostasis rather than from direct radical scavenging. In an in vitro digestion model, loganic acid was completely degraded in the intestinal and colonic fractions [71], and its oral bioavailability in rats was low (2.71–5.58%) [72]. Thus, loganic acid likely undergoes extensive biotransformation after ingestion, and its antioxidant activity may be mediated by its metabolites. However, data on the metabolism of loganic acid are limited, and further studies are needed to clarify this issue. A similar conclusion regarding the relationship between antioxidant activity and chemical composition was reported by Guo et al. [73], who demonstrated that in vitro antioxidant activity depends more strongly on TPC than on iridoid content. They demonstrated that the iridoid fraction of honeysuckle berry extract exhibited lower activity in ABTS, DPPH, and FRAP assays compared with the polyphenol-rich fraction. The greatest difference was observed in the FRAP assay, where the antioxidant activity of the polyphenolic fraction was approximately 8.5-fold higher than that of the iridoid fraction [73].

### 3.5. In Vitro Antidiabetic Activity

The inhibitory effects of the extracts and pure compounds on carbohydrate-digesting enzymes are summarized in Table 4. To the best of our knowledge, there are no studies on the *α*-amylase and *α*-glucosidase inhibitory activity of resin-purified extracts from honeysuckle berries. Inhibitory activity against carbohydrate-digesting enzymes has previously been reported for bilberry (*Vaccinium myrtillus* L.) [74], anthocyanin-rich passion fruit (*Passiflora edulis* Sims) epicarp [75], and cornelian cherry extracts [22]. The bilberry extract purified on Amberlite XAD-7 resin inhibited *α*-glucosidase with an IC_50_ of 0.31 mg/mL, which was approximately 13-fold lower than that of acarbose. In contrast, its inhibitory effect on *α*-amylase was less pronounced; the IC_50_ for *α*-amylase was 4.06 mg/mL, which was more than 200-fold higher than that of acarbose. The honeysuckle berry extract obtained from ripe fruits showed weaker inhibitory activity against carbohydrate-digestive enzymes, and its IC_50_ values were higher than those of acarbose (19-fold higher for *α*-glucosidase and 46-fold higher for *α*-amylase). Because anthocyanins are commonly suggested to act as *α*-amylase and *α*-glucosidase inhibitors, we propose that these differences may be associated with variations in anthocyanin content and profile. The bilberry extract contained delphinidin, malvidin, and petunidin glycosides, whereas cyanidin glycosides were less abundant [74]. In contrast, the anthocyanin profile of honeysuckle berry extract was less diverse, with cyanidin glycosides accounting for 94% of the total anthocyanin content. The extract from passion fruit pericarp purified on Amberlite XAD-16 also contained a mixture of cyanidin and delphinidin glycosides (30.34 mg/g) and exhibited inhibitory activity against carbohydrate-digesting enzymes, although weaker than that observed for honeysuckle berry and bilberry extracts. The IC_50_ values for the passion fruit pericarp extract against *α*-amylase and *α*-glucosidase were 7.99 and 12.80 mg/mL, respectively [75]. Resin-purified extracts from ripe, unripe, and lactofermented cornelian cherry inhibited *α*-amylase more strongly than extracts from honeysuckle berry [22]. The differences in *α*-amylase inhibition among the cornelian cherry extracts were not statistically significant. This may result from the fact that the extract from ripe cornelian cherry fruits contained approximately 60 times less anthocyanins, and their profile differed from that of the unripe fruits. Despite differences in the chemical composition of cornelian cherry extracts (lower content of phenolic acids and higher content of hydrolysable tannins), the extracts inhibited α-glucosidase at a similar level to honeysuckle berry extracts (IC_50_ = 1.78–1.78 mg/mL) [22].

The current study, together with the above-mentioned reports, confirms that anthocyanin-rich extracts may be considered hypoglycemic agents acting through inhibition of enzymes involved in carbohydrate hydrolysis. In the current study, the anthocyanin-rich extract from ripe fruits inhibited *α*-amylase more strongly than the extracts without anthocyanins. All tested extracts inhibited *α*-glucosidase to a similar extent (1.67–1.86 mg/mL). Anthocyanins have been reported as α-amylase and α-glucosidase inhibitors in other plant matrices, our results suggest that, in the studied extracts, the observed inhibitory activity may result from additive or synergistic effects of other compounds, including phenolic acids and flavonols.

Although above mentioned results suggested that the inhibitory effect was associated with the high anthocyanin content, our results demonstrate that extracts from unripe and unripe-fermented fruits, without anthocyanins, inhibit *α*-glucosidase with comparable potency to anthocyanin-rich ripe fruit extracts.

### 3.6. Pearson Correlation and PCA

In the current study, we observed a strong positive correlation between TPC, anthocyanin, and iridoid contents, and antioxidant activity (R = 0.927–1.000) (Table S1). Because a high content of anthocyanins and iridoids is a common characteristic of extracts from ripe fruits, the correlation between iridoid content and antioxidant activity was positive, although loganic acid itself does not exhibit antioxidant activity in in vitro assays. A strong correlation was also found between TPC, anthocyanin content, and *α*-amylase inhibition (R = −0.957 and −0.945, respectively). In contrast, no correlation or only a weak correlation was observed between the sum of groups of biologically active compounds, TPC, antioxidant activity, and *α*-glucosidase inhibition (R = 0.270 to −0.218). It should be noted that, for enzyme inhibition, IC_50_ values were used for the correlation analysis; therefore, negative correlation coefficients indicate a stronger relationship between the analyzed parameters, whereas positive values indicate the absence or weakness of such a relationship.

A strong relationship between total anthocyanin content, antioxidant activity (ABTS, FRAP, and DPPH), and *α*-amylase inhibition is also shown in the PCA plot (Figure S1). A strong relationship between total anthocyanins, total phenolics, and *α*-amylase inhibition has also been reported by Żurek et al. [21], who analyzed the peel and flesh of honeysuckle berries.

## 4. Conclusions

Extracts from ripe, unripe, and lactofermented honeysuckle berries are a source of polyphenols (cinnamic acid derivatives, flavonol glycosides, and flavan-3-ols) and iridoids (loganic acid, loganin derivatives, and sweroside). The extract from ripe honeysuckle berries is characterized by the presence of anthocyanins (cyanidin, pelargonidin, and peonidin derivatives), the highest content of biologically active compounds quantified using HPLC-PDA, as well as the highest TPC measured using the Folin–Ciocalteu method. The strongest antioxidant and antidiabetic activity of the ripe fruit extract may result from its high anthocyanin content, as well as the overall polyphenol content. Weaker antioxidant and *α*-amylase inhibitory activity, as well as comparable *α*-glucosidase inhibitory activity, were observed for extracts from unripe and fermented fruits. Since the pure compounds cyanidin-3-*O*-glucoside and loganic acid did not exhibit antidiabetic activity, the biological properties of the extracts may result from the additive or synergistic effects of compounds present in the extracts. The ripening stage of the fruits used as raw material for extract preparation significantly influenced the chemical composition and biological properties of the extracts. Lactic acid fermentation of unripe fruits with *Levilactobacillus brevis* SBHE2 did not cause significant changes in the chemical composition or biological properties of the extracts. In conclusion, all honeysuckle berry extracts, particularly the extract obtained from unripe fruits, may be considered potential agents in preventing and supporting the treatment of disorders associated with oxidative stress and hyperglycemia.

## Figures and Tables

**Table 1:** Microbiota of products from fermented unripe honeysuckle berry fruits (log CFU/mL).

No.	Variant of Fermented Unripe Honeysuckle Berries	Enterobacteriaceae	Yeasts	Molds	LAB	TMC
1	Fresh fruits + T (HM, SF)	<1	5.70 ± 0.21	<1	<1	6.36 ± 0.17
2	Fresh fruits + S (HM, SF)	<1	5.63 ± 0.36	<1	<1	7.20 ± 0.60
3	Frozen fruits + T (HM, SF)	<1	5.86 ± 0.22	3.30 ± 0.49	<1	6.08 ± 0.50
4	Fresh fruits + T (17 °C, SF)	<1	5.30 ± 0.04	<1	<1	6.54 ± 0.23
5	Fresh fruits + T (17, CF— *Lactiplantibacillus plantarum* ZP71)	<1	5.75 ± 0.21	2.78 ± 0.15	<1	5.15 ± 0.16
6	Fresh fruits + T (17 °C, CF— *Levilactobacillus brevis* SBHE2)	<1	1.48 ± 0.01	<1	7.26 ± 0.28	7.28 ± 0.22

Data are presented as mean of two replicates ± standard deviation. LAB, lactic acid bacteria; TMC, Total Mesophilic Count; T, thyme; S, savory; HM, homemade product; 17 °C, product fermented under controlled temperature at 17 °C; SF, spontaneous fermentation; CF, controlled fermentation.

**Table 2:** UPLC-ESI-qTOF-MS/MS analysis of extracts from ripe, unripe, and unripe lactofermented honeysuckle berry fruits.

No.	tR (min)	MS1 (*m*/*z*)	MS2 Other Ions (*m*/*z*)	Assigned Identification	References	Fruit Extract
Positive ion mode, ESI^+^						
1	3.15	611	449, 287	Cyanidin 3,5-*O*-diglucoside	[7,31,32,33]	RF
2	3.93	449	287	Cyanidin 3-*O*-glucoside	[7,31,32,33]	RF
3	4.15	595	287	Cyanidin 3-*O*-rutinoside	[7,31,32,33]	RF
4	4.50	433	287	Pelargonidin 3-*O*-glucoside	[7,31,32,33]	RF
5	4.81	463	301	Peonidin 3-*O*-glucoside	[7,31,32]	RF
6	5.27	597	303, 257	Delphinidin 3-*O*-sambubioside	[33,34]	RF
Negative ion mode, ESI^−^						
7	3.15	353	191, 179	3-*O*-caffeoylquinic acid (neochlorogenic acid)	[7,31,32,35]	RF, UF, FF
8	3.54	627	303, 285	Taxifolin 7-*O*-dihexoside	[7]	RF
9	3.71	577	289	Procyanidin dimer (1)	[35]	RF, UF, FF
10	3.78	375, 751 [2M − H]^−^	213, 169, 151	Loganic acid	[7,36]	RF, UF, FF
11	4.10	353, 707 [2M − H]^−^	191	5-*O*-caffeoylquinic acid (chlorogenic acid)	[7,31,32,35]	RF, UF, FF
12	4.27	507, 1015 [2M − H]^−^	213, 169, 151, 125	Loganic acid 7-*O*-pentoside	[7,36]	RF, UF, FF
13	4.34	1153	865, 577, 289	Procyanidin tetramer (1)	[7,35]	UF, FF
14	4.55	373	211, 167, 149	Dehydrologanic acid	-	RF, UF, FF
15	4.69	507, 1015 [2M − H]^−^	177, 133, 125, 101	7-epi loganic acid 7-*O*-pentoside	[7,36]	RF, UF, FF
16	4.70	865	577, 289	Procyanidin trimer (1)	[7,35]	RF, UF, FF
17	4.87	367	161	*O*-caffeoylquinic acid methyl ester (1)	[37,38]	RF, UF, FF
18	5.01	353	191	Caffeoylquinic acid	[7]	RF, UF, FF
19	5.08	289	245	Epicatechin	[7,35]	RF, UF, FF
20	5.29	865	577, 289	Procyanidin trimer (2)	[7,35]	UF
21	5.36	567 [M + HCOOH − H]^−^, 521	227, 101	Pentosyl loganin	[7,36]	RF, UF, FF
22	5.50	865	577, 289, 287	Procyanidin trimer (3)	[7,35]	RF, UF, FF
23	5.71	357, 403 [M + HCOOH − H]^−^	149, 125, 101	Sweroside	[7,36]	RF, UF, FF
24	5.74	625, 463, 419	300, 257	Quercetin *O*-dihexoside	[7]	UR, RF
25	5.74	1153	577, 289	Procyanidin tetramer (2)	[7,35]	UF, FF
26	5.88	535	195, 125, 101	Pentosyl sweroside	[36]	RF, UF, FF
27	5.99	457	217, 167, 127, 83	Unidentified iridoid	-	UF
28	6.13	595	301	Quercetin *O*-vicianoside	[7]	RF, UF, FF
29	6.20	367	179, 135	*O*-caffeoylquinic acid methyl ester (2)	[37]	RF, UF, FF
30	6.55	609	301	Quercetin 3-*O*-rutinoside	[7,32,35]	RF, UF, FF
31	6.72	865	577, 289	Procyanidin trimer (4)	[7,35]	UF
32	6.72	593	447, 285	Luteolin 7-*O*-rutinoside	[7,35]	RF, UF, FF
33	6.80	463	301	Quercetin 3-*O*-glucoside	[7,31,35]	RF, UF, FF
34	6.97	609	315	Isorhamnetin hexoside-pentoside	-	FF
35	7.25	593	285	Luteolin 3-*O*-deoxyhexosyl-hexoside	[7]	RF, UF, FF
36	7.38	623	315	Isorhamnetin 3-*O*-rutinoside	-	RF, UF, FF
37	7.49	577	289	Procyanidin dimer (2)	[7,35]	RF, UF, FF
38	7.53	515	353, 191	Dicaffeoylquinic acid (1)	[7,35]	RF, UF, FF
39	7.70	477	315	Isorhamnetin hexoside	-	RF, UF, FF
40	7.74	505	301	Quercetin 3-*O*-acetylhexoside	[7]	UF, FF
41	7.88	607	299	Diosmetin 7-*O*-rutinoside	[7]	RF, UF, FF
42	8.05	515	353	Dicaffeoylquinic acid (2)	[7,35]	RF, UF, FF

RF, ripe fruits; UF, unripe fruits; FF, fermented fruits.

**Table 3:** Phenolic compounds and iridoids quantified by HPLC-PDA in honeysuckle berry extracts [mg/g of extract].

No.	Compound	Ripe Fruit Extract	Unripe Fruit Extract	Fermented Fruit Extract
1	Cyanidin 3,5-*O*-diglucoside	13.71 ± 4.13	n.a.	n.a.
2	Cyanidin 3-*O*-glucoside	239.42 ± 0.32	n.a.	n.a.
3	Cyanidin 3-*O*-rutinoside	20.46 ± 2.24	n.a.	n.a.
4	Pelargonidin 3-*O*-glucoside	4.20 ± 1.72	n.a.	n.a.
5	Peonidin 3-*O*-glucoside	11.93 ± 2.04	n.a.	n.a.
	**Total anthocyanins**	**289.72 ± 9.80**	**-**	**-**
7	3-*O*-Caffeoylquinic acid	3.81 ± 0.03 ^c^	9.41 ± 0.45 ^b^	15.10 ± 0.93 ^a^
11	5-*O*-Caffeoylquinic acid	26.85 ± 0.22 ^c^	65.31 ± 1.49 ^a^	56.10 ± 3.49 ^b^
18	Caffeoylquinic acid	1.66 ± 0.08	t.a.	t.a.
29	*O*-caffeoylquinic acid methyl ester (2)	2.06 ± 2.30 ^a^	1.02 ± 0.07 ^a^	1.06 ± 0.07 ^a^
38	Dicaffeoylquinic acid (1)	2.59 ± 0.02 ^c^	6.23 ± 0.05 ^b^	10.50 ± 0.65 ^a^
42	Dicaffeoylquinic acid (2)	0.39 ± 0.00 ^a^	0.16 ± 0.02 ^b^	0.35 ± 0.03 ^a^
	**Total phenolic acids**	**37.37 ± 2.07 ^b^**	**82.13 ± 2.04 ^a^**	**83.11 ± 5.16 ^a^**
28	Quercetin *O*-vicianoside	0.73 ± 0.08 ^b^	0.92 ± 0.01 ^a^	1.05 ± 0.06 ^a^
30	Quercetin 3-*O*-rutinoside	4.88 ± 0.04 ^b^	5.85 ± 0.49 ^a^	3.18 ± 0.08 ^c^
33	Quercetin 3-*O*-glucoside	17.05 ± 0.15 ^b^	11.18 ± 0.19 ^c^	19.40 ± 1.20 ^a^
34	Isorhamnetin hexoside-pentoside	1.48 ± 0.01 ^a^	0.83 ± 0.01 ^c^	1.27 ± 0.11 ^b^
36	Isorhamnetin 3-*O*-rutinoside	0.74 ± 0.03 ^b^	1.60 ± 0.10 ^a^	1.60 ± 0.10 ^a^
39	Isorhamnetin hexoside	0.90 ± 0.00 ^a^	0.45 ± 0.03 ^b^	1.03 ± 0.08 ^a^
	**Total flavonols**	**25.79 ± 0.15 ^a^**	**20.83 ± 0.83 ^b^**	**27.53 ± 1.65 ^a^**
10	Loganic acid	19.48 ± 0.32 ^b^	39.42 ± 3.55 ^a^	27.67 ± 3.87 ^b^
12	Loganic acid 7-*O*-pentoside	25.29 ± 0.72 ^a^	8.74 ± 0.53 ^b^	3.27 ± 0.49 ^c^
15	7-Epi loganic acid 7-*O*-pentoside	31.70 ± 1.29 ^a^	13.50 ± 0.22 ^b^	16.48 ± 3.22 ^b^
21	Pentosyl loganin	4.69 ± 0.59 ^a^	10.20 ± 1.92 ^a^	7.37 ± 2.26 ^a^
23	Sweroside	55.92 ± 1.87 ^a^	4.25 ± 1.52 ^b^	9.70 ± 3.50 ^b^
26	Pentosyl sweroside	1.22 ± 0.46 ^a^	11.88 ± 1.25 ^a^	1.84 ± 1.76 ^a^
	**Total iridoids**	**138.30 ± 0.30 ^a^**	**88.00 ± 9.00 ^b^**	**66.35 ± 15.10 ^b^**
	**TOTAL**	**491.17 ± 7.22 ^a^**	**190.96 ± 11.85 ^b^**	**176.98 ± 21.88 ^b^**

Data are presented as mean of three replicates ± standard deviation. Letters a–c within columns indicate significant differences (*p* < 0.05; ANOVA, Duncan’s test); n.a., not abundant; t.a., trace amounts.

**Table 4:** Total phenolic content, antioxidant, and antidiabetic activities of freeze-dried extracts from ripe, unripe, and fermented honeysuckle berries.

TPC/ Biological Activity	Ripe Fruit Extract	Unripe Fruit Extract	Fermented Fruit Extract	Loganic Acid	Cyanidin 3-*O*-Glucoside	Acarbose
**TPC** (g GAE/100 g)	39.01 ± 1.61 ^a^	22.91 ± 0.04 ^b^	23.96 ± 0.60 ^b^	−	−	−
**ABTS** (mmol Tx/100 g)	317.50 ± 4.45 ^b^	97.33 ± 0.95 ^c^	97.65 ± 0.37 ^c^	n.a.	749.48 ± 20.02 ^a^	−
**DPPH** (mmol Tx/100 g)	161.17 ± 3.06 ^b^	55.88 ± 1.41 ^c^	54.96 ± 0.55 ^c^	n.a.	439.85 ± 00.00 ^a^	−
**FRAP** (mmol Tx/100 g)	293.88 ± 7.74 ^b^	108.72 ± 2.63 ^c^	112.18 ± 1.12 ^c^	n.a.	567.19 ± 14.52 ^a^	−
***α*-amylase inhibition** (IC_50_ [mg/mL])	2.34 ± 0.03 ^b^	3.46 ± 0.13 ^a^	3.32 ± 0.37 ^a^	n.a.	n.a.	0.05 ± 0.01 ^c^
***α*-glucosidase inhibition** (IC_50_ [mg/mL])	1.86 ± 0.76 ^a^	1.80 ± 0.16 ^a^	1.67 ± 0.19 ^a^	n.a.	n.a.	0.10 ± 0.00 ^b^

Data are presented as mean of two replicates ± standard deviation. Letters a–c within rows indicate significant differences (*p* < 0.05; ANOVA, Duncan’s test). GAE, gallic acid equivalent; n.a., not active; TPC, total phenolic content; Tx, Trolox; −, not applicable.

## Data Availability

The original contributions presented in this study are included in the article/Supplementary Materials. Further inquiries can be directed to the corresponding author.

## Supplementary Materials

The following supporting information can be downloaded at: https://www.mdpi.com/article/10.3390/biom16050673/s1, Figure S1: Principal component analysis of chemical composition, antioxidant activity and glycosidases inhibition. Table S1: Pearson correlation (R) between phenolic compounds, iridoids, antioxidant activity and enzyme inhibition.

## Abbreviations

The following abbreviations are used in this manuscript:

NCDs	Noncommunicable diseases
WHO	World Health Organization
MDA	Malondialdehyde
oxLDL-C	Low-density lipoprotein cholesterol
TAC	Total antioxidant capacity
ABTS	2,2′-Azinobis(3-ethylbenzothiazoline-6-sulfonic acid)
DPPH	1,1-Diphenyl-2-picrylhydrazyl
TPTZ	2,4,6-Tri(2-pyridyl)-s-triazine
LAB	Lactic acid bacteria
TPC	Total phenolic content
Tx	Trolox
PCA	Principal component analysis
CFU	Colony forming unit
TMC	Total mesophilic count
TIC	Total ion chromatogram
fw	Fresh weight
GAE	Gallic acid equivalent
ROS	Reactive oxygen species
STZ	Streptozotocin
GSH	Glutathione
CAT	Catalase
GPx	Glutathione peroxidase
GR	Glutathione reductase
