# Supplementary material for: Unlocking the Functional Potential of Lonicera caerulea: Chemical Profile, Antioxidant, and α-Amylase and α-Glucosidase Inhibitory Activities of Extracts from Ripe, Unripe, and Lactofermented Fruits
Source: Biomolecules. 2026 May 1;16(5):673. doi: 10.3390/biom16050673 (PMC13204703; doi:10.3390/biom16050673)
Supplement: Supplementary file 1 [file biomolecules-16-00673-s001.zip › biomolecules-4262367-supplementary.pdf]

Table S1. Pearson correlation (R) between phenolic compounds, iridoids, antioxidant activity and enzymes inhibition.

	Anthocyanins*	Phenolic acids*	Flavo-nols*	Iridoids*	TPC	ABTS	DPPH	FRAP	α-Amylase inhibition**
**Phenolic acids***	-0.988								
**Flavonols***	0.466	-0.344							
**Iridoids***	0.930	-0.929	0.235						
**TPC**	0.996	-0.972	0.518	0.914					
**ABTS**	0.999	-0.988	0.469	0.930	0.992				
**DPPH**	1.000	-0.988	0.461	0.934	0.995	0.999			
**FRAP**	1.000	-0.985	0.482	0.927	0.997	0.998	1.000		
**α-Amylase inhibition****	-0.945	0.898	-0.575	-0.896	-0.957	-0.945	-0.945	-0.947	
**α-Glucosidase inhibition****	0.200	-0.156	-0.010	0.270	0.252	0.157	0.200	0.207	-0.218

*Sum of compounds calculated using HPLC-PDA method; ** The correlation was calculated for the IC_{50} values.

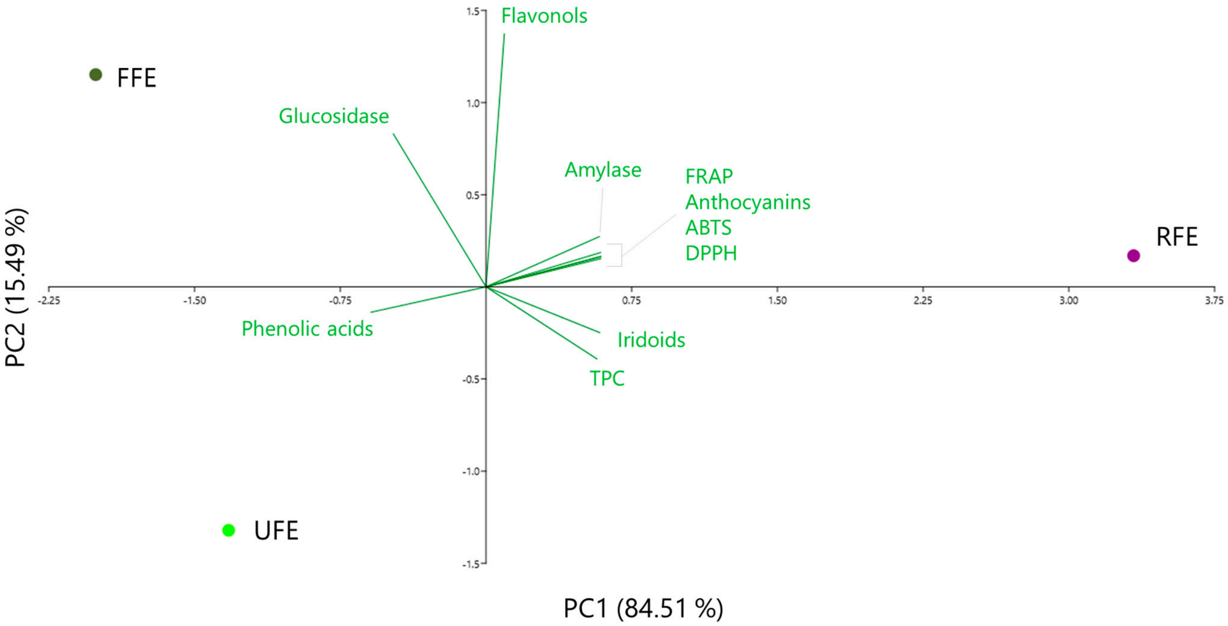

Figure S1. Principal component analysis of chemical composition, antioxidant activity and glycosidases inhibition. Anthocyanins, Phenolic acids, Flavonols and Iridoids refers to the sum of compounds determined using HPLC-PDA. Amylase and Glucosidase refers to inhibition of glycosidases. RFE, Ripe Fruit Extract; UFE, Unripe Fruit Extract; FFE, Fermented Fruit Extract, TPC, Total Phenolic Content.
